# Microbiota profiling with long amplicons using Nanopore sequencing: full-length 16S rRNA gene and the 16S-ITS-23S of the *rrn* operon

**DOI:** 10.12688/f1000research.16817.2

**Published:** 2019-08-01

**Authors:** Anna Cuscó, Carlotta Catozzi, Joaquim Viñes, Armand Sanchez, Olga Francino

**Affiliations:** 1 Vetgenomics, SL, Bellaterra (Cerdanyola del Vallès), Barcelona, 08193, Spain; 2 Dipartimento di Medicina Veterinaria, Università degli Studi di Milano, Milano, Italy; 3 Molecular Genetics Veterinary Service (SVGM), Universitat Autonoma of Barcelona, Bellaterra (Cerdanyola del Vallès), Barcelona, 08193, Spain

**Keywords:** microbiome, microbiota, 16S, rrn operon, nanopore, canine, low-biomass, skin, dog

## Abstract

**Background: **Profiling the microbiome of low-biomass samples is challenging for metagenomics since these samples are prone to contain DNA from other sources (e.g. host or environment). The usual approach is sequencing short regions of the 16S rRNA gene, which fails to assign taxonomy to genus and species level. To achieve an increased taxonomic resolution, we aim to develop long-amplicon PCR-based approaches using Nanopore sequencing. We assessed two different genetic markers: the full-length 16S rRNA (~1,500 bp) and the 16S-ITS-23S region from the *rrn* operon (4,300 bp).

**Methods: **We sequenced a clinical isolate of *Staphylococcus pseudintermedius*, two mock communities and two pools of low-biomass samples (dog skin). Nanopore sequencing was performed on MinION™ using the 1D PCR barcoding kit. Sequences were pre-processed, and data were analyzed using EPI2ME or Minimap2 with *rrn* database. Consensus sequences of the 16S-ITS-23S genetic marker were obtained using canu.

**Results: **The full-length 16S rRNA and the 16S-ITS-23S region of the *rrn* operon were used to retrieve the microbiota composition of the samples at the genus and species level. For the *Staphylococcus pseudintermedius *isolate, the amplicons were assigned to the correct bacterial species in ~98% of the cases with the16S-ITS-23S genetic marker, and in ~68%, with the 16S rRNA gene when using EPI2ME. Using mock communities, we found that the full-length 16S rRNA gene represented better the abundances of a microbial community; whereas, 16S-ITS-23S obtained better resolution at the species level. Finally, we characterized low-biomass skin microbiota samples and detected species with an environmental origin.

**Conclusions: **Both full-length 16S rRNA and the 16S-ITS-23S of the *rrn* operon retrieved the microbiota composition of simple and complex microbial communities, even from the low-biomass samples such as dog skin. For an increased resolution at the species level, targeting the 16S-ITS-23S of the *rrn* operon would be the best choice.

## Introduction

The microbiota profile of low-biomass samples such as skin is challenging for metagenomics. These samples are prone to contain DNA contamination from the host or exogenous sources, which can overcome the DNA of interest ^1, 2^. Thus, the usual approach is amplifying and sequencing certain genetic markers that are ubiquitously found within the studied kingdom rather than performing metagenomics. Ribosomal marker genes are a common choice: 16S rRNA and 23S rRNA genes to taxonomically classify bacteria ^3, 4^; and ITS1 and ITS2 regions for fungi ^5, 6^.

Until now, most studies of microbiota rely on massive parallel sequencing, and target a short fragment of the 16S rRNA gene, which presents nine hypervariable regions (V1-V9) that are used to infer taxonomy ^7, 8^. The most common choices for host-associated microbiota are V4 or V1-V2 regions, which present different taxonomic coverage and resolution depending on the taxa ^9, 10^.

Apart from the biases derived from the primer choice, short fragment strategies usually fail to assign taxonomy reliably at the genus and species level. This taxonomic resolution is particularly useful when associating microbiota to clinics such as in characterizing disease status or when developing microbiota-based products, such as pre- or pro-biotics ^11^. For example, in human atopic dermatitis (AD) the signature for AD-prone skin when compared to healthy skin was enriched for *Streptococcus* and *Gemella*, but depleted in *Dermacoccus*. Moreover, nine different bacterial species were identified to have significant AD-associated microbiome differences ^12^. In canine atopic dermatitis, *Staphylococcus pseudintermedius* has been classically associated with the disease. Microbiota studies of canine atopic dermatitis presented an overrepresentation of *Staphylococcus* genus ^13, 14^, but the species was only confirmed when complementing the studies using directed qPCRs for the species of interest ^13^ or using a *Staphylococcus*-specific database and V1-V3 region amplification ^14^.

With the launching of single-molecule technology sequencers (e.g. PacBio or Oxford Nanopore Technologies), these short-length associated issues can be overcome by sequencing the full-length 16S rRNA gene (~1,500 bp) or even the nearly-complete *rrn* operon (~4,300 bp), which includes the 16S rRNA gene, ITS region, and 23S rRNA gene.

Several studies assessing the full-length 16S rRNA gene have already been performed using Nanopore sequencing to: i) characterize artificial bacterial communities (mock community) ^15– 17^; ii) complex microbiota samples, from the mouse gut ^18^, wastewater ^19^, microalgae ^20^ and dog skin ^21^; and iii) the pathogenic agent in a clinical sample ^22– 24^. Some studies have been performed using the nearly-complete *rrn* operon to characterize mock communities ^25^ and complex natural communities ^26^.

Here, we aim to assess these two long-amplicon approaches using MinION ^TM^ (Oxford Nanopore Technologies), a single-molecule sequencer that is portable, affordable with a small budget and offers long-read output. Its main limitation is a higher error rate than massive sequencing. We will test our approaches by sequencing several samples with different degrees of complexity: i) a clinical isolate of *Staphylococcus pseudintermedius*, ii) two bacterial mock communities; and iii) two complex skin microbiota samples.

## Methods

### Samples and DNA extraction

We first sequenced a pure bacterial isolate of *S. pseudintermedius* obtained from the ear of a dog affected by otitis.

Then, we used two DNA mock communities as simple and well-defined microbiota samples:

- HM-783D, kindly donated by BEI resources, containing genomic DNA from 20 bacterial strains with staggered ribosomal RNA operon counts (between 10 ^3^ and 10 ^6^ copies per organism per μl).
- ZymoBIOMICS™ Microbial Community DNA standard that contained a mixture of genomic DNA extracted from pure cultures of eight bacterial strains.

As a complex microbial community, we used two DNA sample pools from the skin microbiota of healthy dogs targeting two different skin sites: i) dorsal back (DNA from two dorsal samples from Beagle dogs); and ii) chin (DNA from five chin samples from Golden Retriever/Labrador crossed dogs). Skin microbiota samples were collected using Sterile Catch-All™ Sample Collection Swabs (Epicentre Biotechnologies) soaked in sterile SCF-1 solution (50 mM Tris buffer (pH 8), 1 mM EDTA, and 0.5% Tween-20). DNA was extracted from the swabs using the PowerSoil™ DNA isolation kit (MO BIO) and blank samples were processed simultaneously (for further details on sample collection and DNA extraction see 27).

### PCR amplification of ribosomal markers

We evaluated two ribosomal markers in this study: the full-length 16S rRNA gene (~1,500 bp) and the 16S-ITS-23S region of the ribosomal operon ( *rrn)* (~4,300 bp). Before sequencing, bacterial DNA was amplified using a nested PCR, with a first PCR to add the specific primer sets tagged with the Oxford Nanopore universal tag and a second PCR to add the barcodes from the PCR barcoding kit (EXP-PBC001) (Supplementary Table 1). Each PCR reaction included a no-template control sample to assess possible reagent contamination.

For the first PCR, we targeted: i) the full-length 16S rRNA gene using 16S-27F ^28^ and 16S-1492R ^29^ primer set and ii) the 16S-ITS-23S of the *rrn* operon using 16S-27F and 23S-2241R ^28^ primer set (Supplementary Table 1). All the three primers contained the Oxford Nanopore tag, which is an overhang that allows barcoding the samples during the second PCR.

PCR mixture for the full-length 16S rRNA gene (25 μl total volume) contained 5 ng of DNA template (or 2.5 μl of unquantifiable initial DNA), 1X Phusion® High Fidelity Buffer, 0.2 mM of dNTPs, 0.4 μM of 16S-27F, 0.8 μM of 16S-1492R and 0.5 U of Phusion® Hot Start II Taq Polymerase (Thermo Scientific, Vilnius, Lithuania). The PCR thermal profile consisted of an initial denaturation of 30 s at 98°C, followed by 25 cycles of 15 s at 98°C, 15 s at 51°C, 45 s at 72°C, and a final step of 7 min at 72°C.

PCR mixture for the 16S-ITS-23S of the *rrn* operon (50 μl total volume) contained 5 ng of DNA template (or 2.5 μl of unquantifiable initial DNA), 1X Phusion® High Fidelity Buffer, 0.2 mM μl dNTPs 1 μM each primer and 1 U Phusion® Hot Start II Taq Polymerase. The PCR thermal profile consisted of an initial denaturation of 30 s at 98°C, followed by 25 cycles of 7 s at 98°C, 30 s at 59°C, 150 s at 72°C, and a final step of 10 min at 72°C.

The amplicons were cleaned-up with the AMPure XP beads (Beckman Coulter) using a 0.5X and 0.45X ratio for the 16S rRNA gene and the 16-ITS-23S of the *rrn* operon, respectively. Then, they were quantified using Qubit™ fluorometer (Life Technologies, Carlsbad, CA) and the volume was adjusted to begin the second round of PCR with 0.5 nM of the first PCR product or the complete volume when not reaching the required DNA mass (mostly in the samples that amplified with the 16S-ITS-23S genetic marker).

PCR mixture for the barcoding PCR (100 μl total volume) contained 0.5 nM of the first PCR product (50 ng for the 16S rRNA gene and 142 ng for the 16S-ITS-23S), 1X Phusion® High Fidelity Buffer, 0.2 mM μl dNTPs, and 2 U Phusion® Hot Start II Taq Polymerase. Each PCR tube contained the DNA, the PCR mixture and 2 μl of the specific barcode. The PCR thermal profile consisted of an initial denaturation of 30 s at 98°C, followed by 15 cycles of 7 s at 98°C, 15 s at 62°C, 45 s (for the 16S rRNA gene) or 150 s (for *rrn* operon) at 72°C, and a final step of 10 min at 72°C.

Again, the amplicons were cleaned-up with the AMPure XP beads (Beckman Coulter) using a 0.5X and 0.45X ratio for the 16S rRNA gene and the whole *rrn* operon, respectively. For each sample, quality and quantity were assessed using Nanodrop and Qubit™ fluorometer (Life Technologies, Carlsbad, CA), respectively. The samples with higher DNA concentrations were checked by agarose gel to see the size profile of the PCR products (Supplementary Figure 1).

The different barcoded samples were pooled in equimolar ratio to obtain a final pool (1,000–1,500 ng in 45 μl) to do the sequencing library. In few cases, 16S-ITS-23S amplicons did not reach the initial amount of required DNA and we proceeded with lower input material.

### Nanopore sequencing library preparation

The Ligation Sequencing Kit 1D (SQK-LSK108; Oxford Nanopore Technologies) was used to prepare the amplicon library to load into the MinION ^TM^ (Oxford Nanopore Technologies), following the manufacturer’s protocol. Input DNA samples were composed of 1–1.5 μg of the barcoded DNA pool in a volume of 45 μl and 5 μl of DNA CS (DNA from lambda phage, used as a positive control in the sequencing). The DNA was processed for end repair and dA-tailing using the NEBNext End Repair/dA-tailing Module (New England Biolabs). A purification step using 1X Agencourt AMPure XP beads (Beckman Coulter) was performed.

For the adapter ligation step, a total of 0.2 pmol of the end-prepped DNA were added in a mix containing 50 μl of Blunt/TA ligase master mix (New England Biolabs) and 20 μl of adapter mix and then incubated at room temperature for 10 min. We performed a purification step using Adapter Bead Binding buffer (provided in the SQK-LSK108 kit) and 0.5X Agencourt AMPure XP beads (Beckman Coulter) to finally obtain the DNA library.

We prepared the pre-sequencing mix (14 μl of DNA library) to be loaded by mixing it with Library Loading beads (25.5 μl) and Running Buffer with fuel mix (35.5 μl). We used two SpotON Flow Cells Mk I (R9.4.1) (FLO-MIN106). After the quality control, we primed the flowcell with a mixture of Running Buffer with fuel mix (RBF from SQK-LSK108) and Nuclease-free water (575 μl + 625 μl). Immediately after priming, the nanopore sequencing library was loaded in a dropwise fashion using the SpotON port.

Once the library was loaded, we initiated a standard 48 h sequencing protocol using the MinKNOW™ software v1.15.

### Data analysis workflow

The samples were run using the MinKNOW software. After the run, fast5 files were base-called and de-multiplexed using Albacore v2.3.1. A second de-multiplexing round was performed with Porechop v0.2.3 ^30^, where only the barcodes that agreed with Albacore were kept. Porechop was also used to trim the barcodes and the adapters from the sequences, as well as 45 extra base pairs from each end that correspond to the length of the universal tags and custom primers (See Supplementary Figure 2 for a schematic overview of the process and Supplementary File 1 for the bioinformatics workflow of the mapping approach).

After the trimming, reads were selected by size: 1,200 bp to 1,800 bp for 16S rRNA gene; and 3,500 to 5,000 bp for the 16S-ITS-23S of the *rrn* operon. Afterwards, we removed chimeras with the following approach: i) we mapped each mock community to its mock database and the complex samples to the complete *rrn* database using Minimap2 v2.16 (with base-level alignment and z-score set to 70) ^31^; ii) chimeras were detected and removed using yacrd v0.5 ^32^.

To assign taxonomy to the trimmed and filtered reads we used to strategies: 1) a mapping-based strategy using Minimap2 v2.16 ^31^ (with base-level alignment and z-score set to 70); or 2) a taxonomic classifier using What’s in my Pot (WIMP) ^33^, a workflow from EPI2ME in the Oxford Nanopore Technologies cloud (based on Centrifuge software ^34^).

For the mapping-based strategy, we performed Minimap2 again with the non-chimeric sequences. We applied extra filtering steps to retain the final results: we kept only those reads that aligned to the reference with a block equal or larger than 1,000 bp (for 16S rRNA gene) and 3,000 bp (for the 16S-ITS-23S of the *rrn* operon). For reads that hit two or more references, only the alignments with the highest Smith-Waterman alignment score (AS score) were kept.

The reference databases used in this study were:

- Mock DB: a collection of the complete genomes that were included in each mock community, as described by the manufacturer. The HM-783D database was retrieved from NCBI using the reference accession numbers, while Zymobiomics mock community has already its database online on the Amazon AWS server.
- *rrn* DB: sequences from the whole ribosomal operon from 22,351 different bacterial species retrieved from Genbank by Benitez-Paez *et al.* ^25^. We have manually added a sequence of the *rrn* operon from *S. pseudintermedius*.

For assessing the mapping-based strategy, we have made a subset with the *rrn* DB to exclude all the operons that were representatives of Gammaproteobacteria class as an example to see how the alignment-based approach performs when missing main references within the database. These operons were identified by introducing a list of all the genera of the *rrn* DB as a batch in NCBI Taxonomy browser. The sequences belonging to Gammaproteobacteria (code 1236) were removed from the *rrn* DB.

For the taxonomic classification using the WIMP workflow, which uses the NCBI database, only those hits with a classification score >300 were kept ^34^.

Ampvis2 package in R was used to plot the heatmaps ^35^ and the Phyloseq package, to plot the alpha rarefaction curves ^36^.

An earlier version of this article can be found on bioRxiv (doi: https://doi.org/10.1101/450734)

## Results

We have assessed the performance of the full-length 16S rRNA and the 16S-ITS-23S rRNA region of the ribosomal ( *rrn*) operon to profile the microbial composition of several samples: a bacterial isolate, two mock communities and two complex skin samples (chin and dorsal back).

The samples amplified using the full-length 16S rRNA gene recovered a higher percentage of reads after the quality control when compared to 16S-ITS-23S of the *rrn* operon: 73–95% vs. 30–79%. For the 16S-ITS-23S of the *rrn* operon, the largest percentage of reads was lost during the length trimming step since some of the reads presented lengths that were shorter than expected (Supplementary Table 2).

### Bacterial isolate analysis

We first sequenced an isolate of *S. pseudintermedius* obtained from a canine otitis. When using WIMP approach with the 16S-ITS-23S of the *rrn* operon, 97.5% of the sequences were correctly assigned at the species level as *S. pseudintermedius*. However, with the full-length 16S rRNA gene, 68% of the sequences were correctly assigned at the species level as *S. pseudintermedius,* while 13% at the genus one and ~20% were wrongly assigned, either by not reaching the species level or by giving an incorrect species ( Table 1).

**Table 1.** Taxonomy assignments of *S. pseudintermedius* isolate. Taxonomic assignments were obtained i) using WIMP workflow with NCBI RefSeq database; ii) Minimap2 with *rrn* DB; and iii) Minimap2 with *rrn* DB including *S. pseudintermedius*.

	WIMP (NCBI RefSeq DB)		Minimap2 ( *rrn* DB)		Minimap2 ( *rrn* DB + *S. pseudintermedius*)	
Taxonomy	16S	16S-ITS-23S	16S	16S-ITS-23S	16S	16S-ITS-23S
*Staphylococcus* *pseudintermedius*	68.1%	97.6%	-	-	97.9%	97.5%
*Staphylococcus sp*	13.1%	0.3%	-	-	-	-
Staphylococcus schleiferi *	2.2%	0.3%	94.9%	82.1%	1.94%	2.2%
Staphylococcus aureus *	3.2%	0.2%	0.0%	0.0%	0.0%	0.0%
Staphylococcus lutrae *	2.7%	0.1%	-	-	-	-
Staphylococcus hyicus *	0.3%	0.1%	3.2%	8.2%	0.0%	0.2%
Staphylococcus agnetis *	0.2%	0.0%	1.6%	9.7%	0.0%	0.1%
Other Staphylococcus *	3.7%	1.4%	0.3%	0.0%	0.2%	-
Other species *	6.5%	0.1%	-	-	-	-

*Incorrect bacterial species assignment

When using the mapping approach with the *rrn* DB, we obtained no hit to *S. pseudintermedius*. Instead, they were hitting mostly to *Staphylococcus schleiferi*, which is a closely related species; there were also few hits to *Staphylococcus hyicus* and *Staphylococcus agnetis*. This result was due to the *rrn* DB did not contain any representative of *S. pseudintermedius*. When including *S. pseudintermedius* sequence to the *rrn* DB ( *rrn* + *S. pseudintermedius*) both markers retrieved the correct result with more than 97% of the assignments hitting the correct reference.

**Figure 1.**
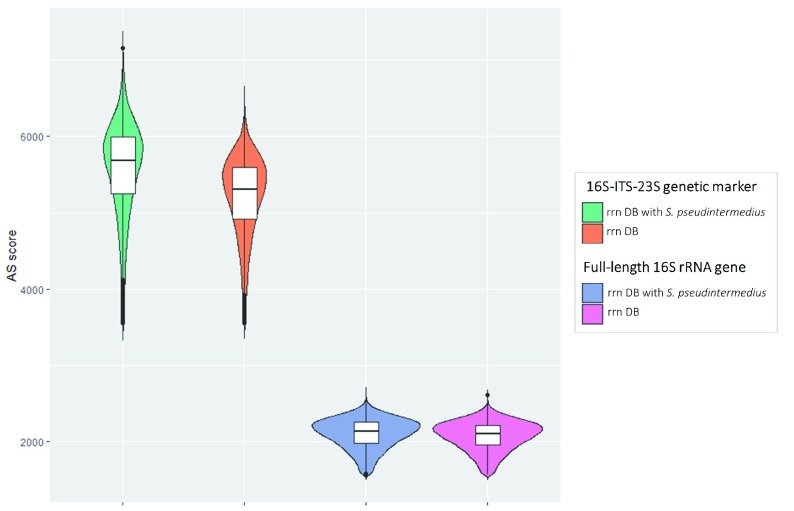
Violin plot representing the distribution of AS score for *S. pseudintermedius* isolate. Alignment scores for each genetic marker were obtained using Minimap2 and either *rrn* DB or *rrn* DB with a reference added for *S. pseudintermedius*. The first two plots are for 16S-ITS-23S, whereas the second ones are for 16S rRNA.

When comparing the alignment results obtained with Minimap2 and *rrn* DB vs *rrn* DB + *S. pseudinteremedius*, we found that the Smith-Waterman alignment score (AS) presented higher values in the correct alignments, especially when using 16S-ITS-23S marker gene ( Figure 1). Thus, the AS score could be a filter to identify a wrong taxonomic assignment due to the lack of a reference in the database.

### Assessment of the mapping-based strategy

As we have already seen for *S. pseudintermedius*, in the mapping strategy a lack of a reference in the database led to an incorrect taxonomic assignment. Since both marker genes chosen for the microbiota profiling are highly conserved among bacteria, the mapping strategy (through Minimap2) will always align to some reference.

To check the behavior of the mapping approach when using an incomplete database, we have performed an example test using the mock communities. We have mapped the mock communities both against the complete *rrn* DB and against a subset of the *rrn* DB without any representative of the Gammaproteobacteria class. The *rrn* DB ^25^ contains 22,351 different bacterial species, including representatives of the species in both mock communities. We have chosen Gammaproteobacteria because each mock community contains three Gammaproteobacteria species, representing around 24% of the total microbial composition.

**Figure 2.**
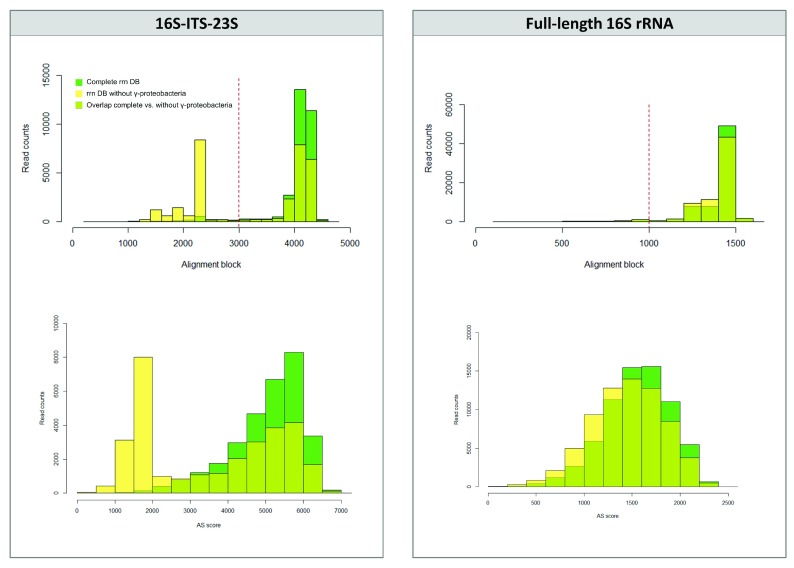
Assessment of the mapping-based strategy using a sample of Zymobiomics mock community. Top part, histograms of the read counts distributed by alignment block depending on the database used (complete *rrn* DB vs *rrn* DB without Gammaproteobacteria). Alignment threshold at 3,000 bp (for 16S-ITS-23S) and 1,000 (for 16S rRNA gene) are marked with a red dashed line. Below part, histograms of the read counts distributed by AS scores depending on the database used (complete *rrn* DB vs *rrn* DB without Gammaproteobacteria).

We checked the alignment score values and the alignment block length to detect any differences on the alignment performance when using complete or an incomplete database. We plotted two histograms: i) read counts distributed by the alignment block length; and ii) read counts distributed by the alignment score (AS) ( Figure 2). For the 16S-ITS-23S genetic marker, we detected a clear pattern: when aligning to the *rrn* DB without Gammaproteobacteria, both histograms changed from a left-skewed distribution to a bimodal distribution with two peaks ( Figure 2). A new peak appeared at the lower values that included the wrong taxonomic assignments, which are species not present in the mock community or the non-concordant hits when compared to the complete *rrn* DB results. Thus, for the 16S-ITS-23S marker, the initial filtering step by alignment block length will get rid of most of the incorrect taxonomic assignments. However, this pattern was not observed with the full-length 16S rRNA gene ( Figure 2) or when closely-related references were present in the database, as seen above for the *S. pseudintermedius* isolate. So, to further confirm taxonomic results (especially for the 16S rRNA gene), we assigned taxonomy using two different bioinformatics approaches that work with different databases.

### Mock community analyses

We analyzed two microbial mock communities to validate the ability of the presented approach: i) to quantify what is expected and detect biases of the technique; and ii) to reach a reliable taxonomic assignment at the species level.

For the first aim, we used the HM-783D mock community that contained genomic DNA from 20 bacterial strains with staggered ribosomal RNA operon counts (from 10 ^3^ to 10 ^6^ copies per organism per µl). This mock community would allow us determining if our approach reliably represents the actual bacterial composition of the community, especially considering the low-abundant species. We assigned taxonomy with Minimap2 and a database containing only the 20 representative bacterial genomes in the HM-783D mock community (Minimap2-mock DB).

**Figure 3.**
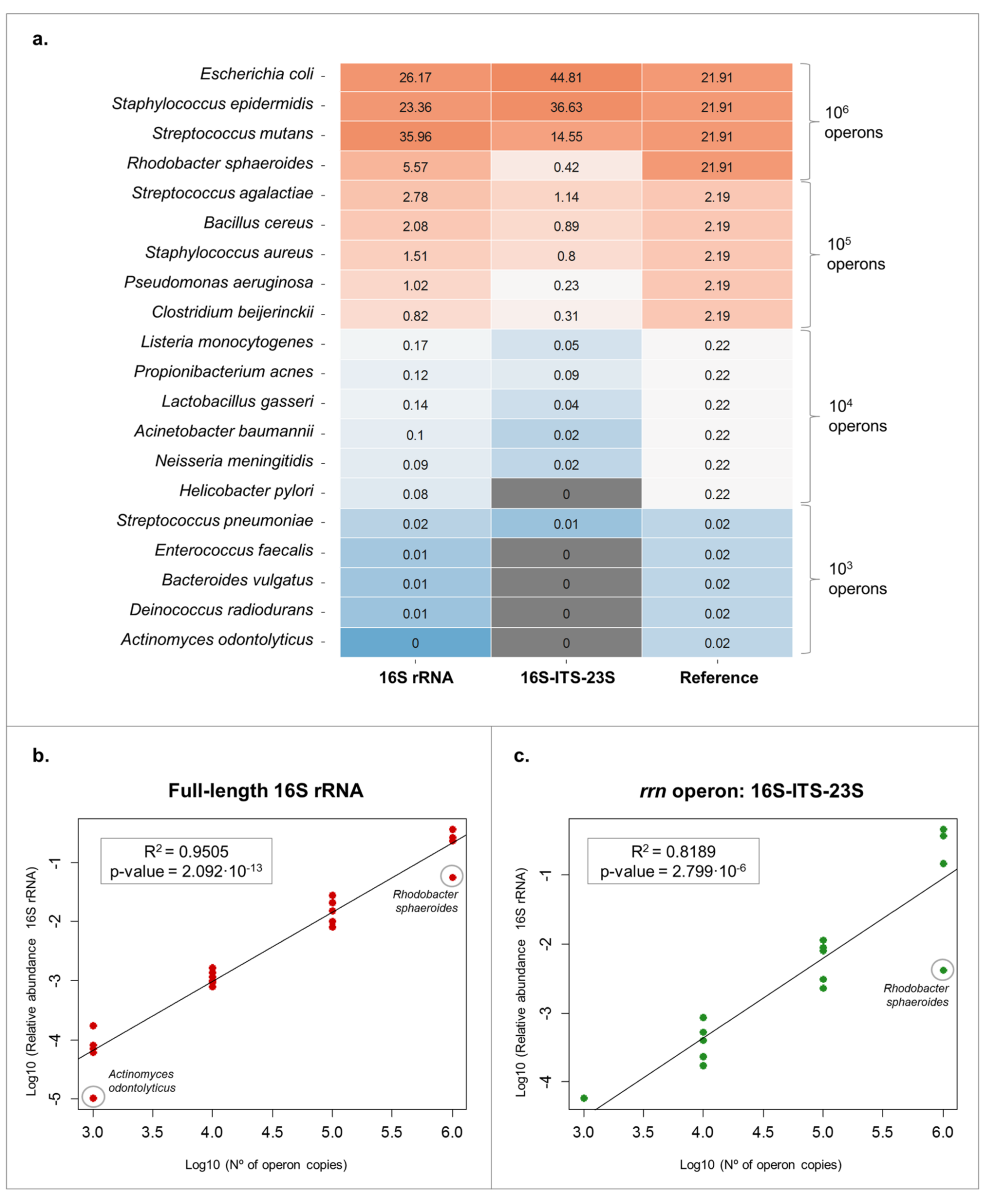
HM-783D mock community analysis. ( **A**) Heat map representing the HM-783D mock community composition when mapped to its mock database. Grey colour represents the bacteria that were not detected (<10 ^4^ copies with *rrn* operon). ( **B**) Linear regression analysis of relative read proportions obtained using full-length 16S rRNA gene for all bacterial species present in HM-783D mock community and the actual operon copies (in log scale). ( **C**) Linear regression analysis of relative read proportions obtained using the 16S-ITS-23S genetic marker for all bacterial species present in HM-783D mock community and the actual operon copies (in log scale).

On the one hand, using the full-length 16S rRNA gene we detected all the bacterial species present in the mock community, even the low-abundant ones. On the other hand, using the 16S-ITS-23S of the *rrn* operon we detected only the most abundant species (at least 10 ^4^ operon copies) ( Figure 3A). This could be due to the lower sequencing depth obtained with 16S-ITS-23S when compared with 16S rRNA (Supplementary Table 2). Moreover, the relative abundances of 16S-ITS-23S sequences were more biased than those obtained from 16S rRNA gene sequencing, which confirmed that the primers for 16S-ITS-23S of the *rrn* operon need to be improved for a better representation of the actual abundances ( Figure 3B and 3C).

To assess if the technique and the analyses would give a reliable taxonomy at the species level we used Zymobiomics mock community, which contains equal quantities of 8 bacterial species. The expected 16S rRNA gene content for each representative is also known, so we were able to determine if the different analysis approaches reliably represented the actual bacterial composition of the community. We sequenced the Zymobiomics mock community twice per marker gene and found that the replicates presented equivalent results.

We assigned taxonomy with three different approaches: i) Minimap2 and a database containing only the 8 bacterial species of the correspondent mock community (mock DB); ii) Minimap2 and a database containing sequences for the *rrn* operon of 22,351 different bacterial species ( *rrn* DB (25)) and iii) WIMP from EPI2ME and NCBI RefSeq database.

**Figure 4.**
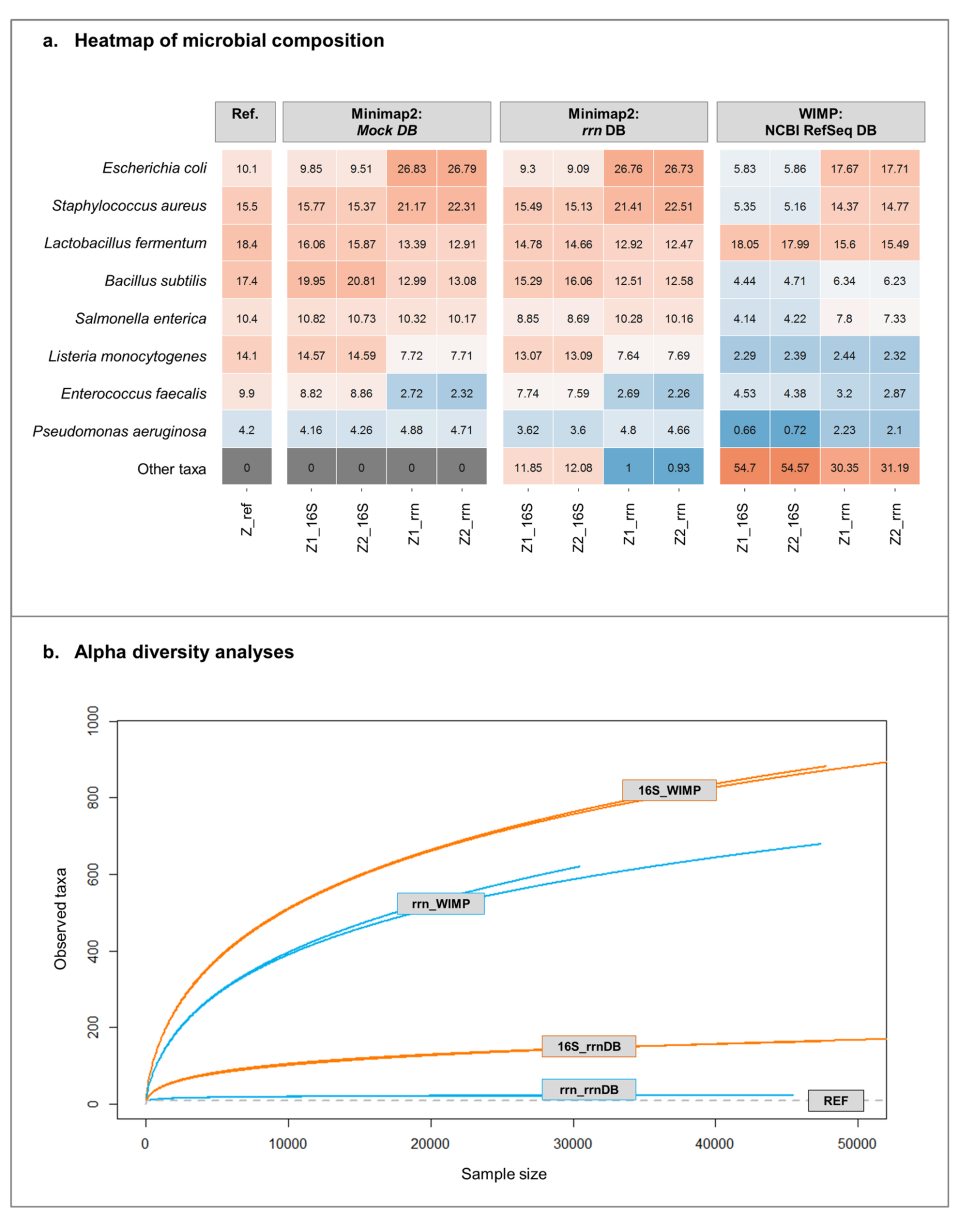
Zymobiomics mock community taxonomic analysis and diversity. ( **A**) Heat map representing the relative abundance of the Zymobiomics mock community. “REF” column represents the theoretical composition of the mock community regarding the 16S rRNA gene content of each bacterium. ( **B**) Alpha diversity rarefaction plot using observed taxa metrics.

Similarly to what we have seen for the HM-783D mock community, when using the mapping strategy with Minimap2 and the mock database, we detected that the full-length 16S rRNA gene retrieved better the actual abundances of the mock community. The 16S-ITS-23S genetic marker over- and underrepresented most of the bacterial species in the mock community. When using larger databases such as *rrn* DB and NCBI RefSeq, both the full-length 16S rRNA gene and the 16S-ITS-23S were able to detect 8 out of 8 bacterial species of the Zymobiomics mock community ( Figure 4A). However, we also detected other taxa that included mostly higher taxonomic rank taxa (sequences not assigned to species level), but also not expected taxa (wrongly-assigned species), especially with WIMP and NCBI RefSeq database (see Supplementary Table 3 for complete taxonomic assignments).

On one hand, the mapping approach using the *rrn* DB provided highly similar results to the reference, despite the larger size of this database ( Figure 4A), especially with 16S-ITS-23S marker (99% of the reads were correctly assigned at the species level with 16S-ITS-23S; near 90% for 16S). On the other hand, with the WIMP workflow and NCBI RefSeq database, a larger number of sequences are classified as “Other taxa”. Again, this is especially remarkable when using the full-length 16S rRNA gene, with > 50% of the taxonomic assignments not hitting the expected bacterial species. The results were also confirmed by alpha diversity analyses: WIMP strategy overestimated the actual bacterial diversity, when compared to *rrn* DB and the reference ( Figure 4B).

### Complex microbial community analyses

We profiled two complex and uncharacterized microbial communities from dog skin (chin and dorsal). We used both long-amplicon markers and the two bioinformatics approaches –Minimap2 and *rrn* DB and WIMP with NCBI RefSeq database– to corroborate the results.

**Figure 5.**
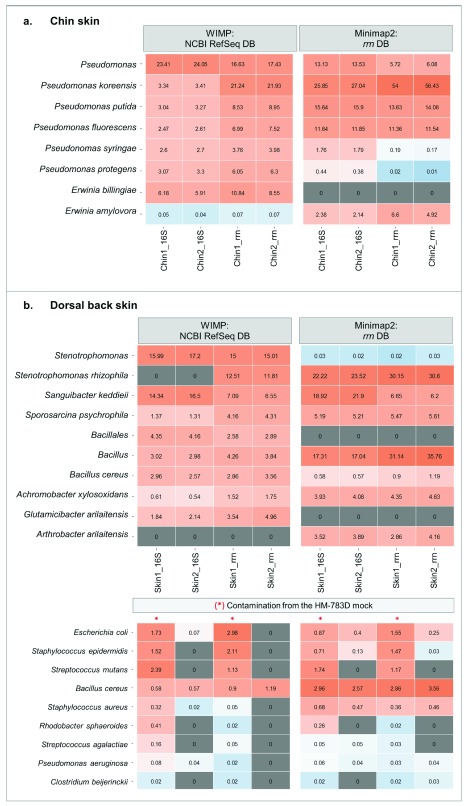
Microbiota composition of complex communities: skin samples of healthy dogs. ( **A**) Chin samples: heat map representing the relative abundance of the main bacterial species in chin samples using WIMP and Minimap2. ( **B**) Dorsal skin samples: heat map representing the relative abundance of the main bacterial species using WIMP and Minimap2. The lower heat map represents the remaining contamination from the previous run using HM-783D mock community within the same flowcell. Samples marked with a red line shared barcode with the mock community.

For chin samples of healthy dogs, we found a high abundance of *Pseudomonas* species (>40% of total relative abundance using 16S rRNA and >60% using 16S-ITS-23S) followed by other genus with lower abundances such as *Erwinia* and *Pantoea*. Focusing on *Pseudomonas*, at the species level we were able to detect that the most abundant species was *Pseudomonas koreensis*, followed by *Pseudomonas putida* and *Pseudomonas fluorescens* ( Figure 5A and Supplementary Table 3). On the other hand, dorsal skin samples were dominated by bacteria from the genera *Stenotrophomonas*, *Sanguibacter*, and *Bacillus*. We reached species level for *Stenotrophomonas rhizophila* and *Sanguibacter keddieii*. It should be noted that *Glutamicibacter arilaitensis* is the same species as *Arthrobacter arilaitensis*, with newer nomenclature ( Figure 5B and Supplementary Table 3). For both skin sample replicates, the results of the most abundant species converged using the two different methods and allowed for characterizing this complex low-biomass microbial community at the species level.

Finally, analyzing the dorsal skin samples, we also detected the presence of contamination from the previous nanopore run (Supplementary Table 2). We sequenced dorsal skin samples twice: one with a barcode previously used for sequencing the HM-783D mock community and another one with a new barcode. We were able to detect mock community representatives within the re-used barcode ( Figure 5B). Some of them were found only in the sample that was using the re-used barcode (Sample_1); others were also present in the skin sample, such as *Bacillus cereus* or *Staphylococcus aureus*. In total, this contamination from the previous run was representing ~6% of the sample composition.

### *De novo* assembly of the 16S-ITS-23S genetic marker

To further confirm the results obtained with the complex samples using directly the raw reads, we performed the assembly and consensus of the 16S-ITS-23S genetic marker using canu and we assigned taxonomy of the consensus sequences using BLAST.

For the HM-783D mock community, we were able to retrieve some of the most abundant bacterial species blasting with >99% of identity to their reference ( *Escherichia coli*, *Staphylococcus epidermidis*, *Streptococcus mutans* and *Clostridium beijerinnkii*). For the Zymobiomics mock community, we found a consensus sequence for all the bacterial species with >99% of identity. The only two exceptions were *Salmonella enterica* that presented a consensus sequence with an identity of 98.8% and *Escherichia coli* that presented no consensus sequence ( Table 2 and Supplementary Table 4).

**Table 2.** Zymobiomics contigs for the 16S-ITS-23S genetic marker and their taxonomic assignment through obtained through blasting.

Contig	Length	n° of reads	covStat	NCBI name	NCBI Accession	% Query coverage	e-value	% identity
tig00000001	4,346	10,900	13,568.84	*Salmonella enterica*	CP012344.2	99%	0.0	98.8%
tig00000003	3,972	2,237	15,554.25	*Bacillus subtilis*	CP002183.1	99%	0.0	99.4%
tig00000004	4,253	7,519	17,430.61	*Staphylococcus aureus*	CP029663.1	99%	0.0	99.1%
tig00000007	4,188	1,588	19,724.96	*Pseudomonas* *aeruginosa*	CP032257.1	100%	0.0	99.5%
tig00000009	4,061	645	18,811.23	*Enterococcus faecalis*	CP025021.1	100%	0.0	99.1%
tig00000010	4,064	1,107	17,360.31	*Listeria monocytogenes*	CP035187.1	99%	0.0	99.2%
tig00000012	4,019	1,347	16,386.90	*Lactobacillus fermentum*	CP034099.1	100%	0.0	99.4%

For the complex microbial communities, the *de novo* consensus sequences usually presented lower identity than those of the mock community. For the dorsal skin, the ones with higher consensus accuracy were *Stenotrophomonas rhizophila* (99.3 %), *Streptococcus mutans* (99.1 %) and *Arthrobacter arilaitensis* (98.6 %), thus confirming the results retrieved using directly the raw reads. For the chin, the three contigs with higher consensus accuracy hit *Pseudomonas fluorescens* (>98.5%). We detected also other contigs with lower consensus accuracy values, with previously not seen taxonomic assignments (Supplementary Table 4).

## Discussion

The full-length 16S rRNA and the 16S-ITS-23S of the *rrn* operon identified the bacterial isolate and revealed the microbiota composition of the mock communities and the complex skin samples, even at the genus and the species level. So, we present this long-amplicon approach as a method to profile the microbiota of low-biomass samples at deeper taxonomic levels.

The long amplicons were analyzed as raw reads (uncorrected), using both a mapping-based approach through Minimap2 and the “What’s in My Pot” workflow by Oxford Nanopore to confirm results using a double approach. Although Nanopore sequencing has a high error rate (average accuracy for the *S. pseudintermedius* isolate: 89%), we compensated this low accuracy with longer fragments to assess the taxonomy of several bacterial communities. In general, the longer the marker, the higher the taxonomical resolution with both analyses performed. In this case, the longer 16S-ITS-23S marker remarkably improved the taxonomy assignment at the species level.

Moreover, we also performed de novo assembly of the 16S-ITS-23S amplicons obtaining consensus sequences that allowed us to validate some of the taxonomy retrieved with the long-amplicon raw reads. A de novo approach allowed retrieving consensus sequences with high accuracy (>99% of identity) for simple microbial communities. However, when working with more complex communities this consensus accuracy was generally lower. These lower accuracies found in complex microbial communities could be due to the lower sequencing depth, an uneven distribution of the bacterial species, and a mix of some closely similar species within the same contig.

Mock communities’ analyses allowed us assessing the performance and the biases of the methodology, from the lab bench to the bioinformatics analyses and final results. In general, we found that the full-length 16S rRNA gene represents better the abundances of a microbial community; whereas, 16S-ITS-23S obtains better resolution at the species level.

So, do the long-amplicon approaches represent the actual bacterial composition? On one hand, we detected biases of our primer sets for both genetic markers, since some of the species of the mock communities were over- and under-represented. For example, *Actinomyces odontolyticus* and *Rhodobacter sphaeroides* seem to not amplify properly, neither with 16S rRNA gene, nor the 16S-ITS-23S of the *rrn* operon. Previous studies also detected the same pattern for these specific bacteria even when using or comparing different primer sets ^16, 21^. Overall, the 16S rRNA primer set seemed less biased than the 16S-ITS-23S of the *rrn* operon, which over- and underrepresented most of the bacterial species, suggesting that the 16S-ITS-23S primers should be improved for unbiased representation of the community.

On the other hand, with the HM-783D staggered mock community –with some low-abundant species– we aimed to assess the sensitivity of both approaches. With the 16S rRNA marker gene, we detected all bacterial members of both mock communities. However, when using the 16S-ITS-23S of the *rrn* operon, some of the low-abundant species in the HM-783D mock community were not detected. This was probably due to the fact that we obtained a lower number of reads –up to one magnitude less than with the 16S rRNA gene. Since we combined 16S rRNA and 16S-ITS-23S amplicons in the same run, this led to an underrepresentation of the 16S-ITS-23S amplicons and consequently a lower sequencing depth. This was probably due to the combination of various issues: i) not enough DNA mass to begin with the indicated number of molecules; ii) reads with shorter size than expected (~1,500 bp); iii) shorter fragments tend to be sequenced preferentially with Nanopore sequencing. Thus, for future studies our recommendation would be multiplexing samples with the same amplicon size to avoid underrepresentation of the longest one and improving PCR parameters or adding more PCR cycles to the longer amplicons to get more input DNA mass.

In the bioinformatics analyses, our aim was to confirm the results with two independent workflows and different databases rather than comparing them. We saw that the most abundant species were usually concordant with both strategies at a qualitative level. Some exceptions were due to the lack of that species in the *rrn* database, such as that seen for *S. pseudintermedius*. With the WIMP workflow and the 16S rRNA gene, many sequences did not reach species level. Previous studies analyzing the microbiota obtained with Nanopore reads have compared the performance of several databases using the 16S-ITS-23S ^26^ and software for the 16S rRNA gene ^22^. They also found similar results as reported here: some false positives associated to specific software ^22^, as well as a high impact on the unclassified reads depending on the size of the database used ^26^.

When using EPI2ME (WIMP with NCBI Ref database), the amplicons from the *S. pseudintermedius* isolate were assigned to the correct bacterial species in ~98% and ~68% of the cases, using the 16S-ITS-23S of the *rrn* operon and 16S rRNA gene, respectively. In a previous study, Moon and collaborators used the full-length 16S rRNA gene for characterizing an isolate of *Campylobacter fetus* and the marker correctly assigned the species for ~89% of the sequences using EPI2ME ^23^. The ratio of success on the correct assignment at the species level depends on the species itself and its degree of sequence similarity in the selected genetic marker. Within the *Staphylococcus* genus, the 16S rRNA gene presents the highest similarity (around ~97%) when compared to other genetic markers ^37^.

On the other hand, we observed that the mapping strategy (through Minimap2) could lead to a wrong assigned species if the interrogated bacterium is not represented on the chosen database. Minimap2 provides faster results than EPI2ME, but it needs an accurate comprehensive and representative database. Extra filtering steps using the alignment block length or Smith-Waterman alignment score could potentially be used to discard a wrong taxonomic assignment.

Switching to complex microbial communities, we found that dog chin was colonized by different *Pseudomonas* species. Recently, Meason-Smith and collaborators found *Pseudomonas* species associated with malodor in bloodhound dogs ^38^. However, these were not the main bacteria found within the skin site tested, but were in low abundance, differing from what we have found here. On the other hand, Riggio and collaborators detected *Pseudomonas* as one of the main genera in canine oral microbiota in the normal, gingivitis and periodontitis groups ^39^. However, the *Pseudomonas* species were not the same that we have detected here. It should be noted that we had characterized these chin samples with 16S V1-V2 amplicons in a previous study ^27^, where we found some mutual exclusion patterns for *Pseudomonadaceae* family. This taxon showed an apparent “invasive pattern”, which could be mainly explained for the recent contact of the dog with an environmental source that contained larger bacterial loads before sampling ^27^. Thus, our main hypothesis is that the *Pseudomonas* species detected on dog chin came from the environment, since they have been previously isolated from environments such as soil or water sources ^40, 41^.

None of the most abundant species in dog dorsal skin had previously been associated with healthy skin microbiota either in human or in dogs. Some of them have an environmental origin, such as *Stenotrophomonas rhizophila*, which is mainly associated with plants ^42^; or *Sporosarcina psychrophila,* which is widely distributed in terrestrial and aquatic environments ^43^. The *Bacillus cereus* main reservoir is also the soil, although it can be a commensal of root plants and guts of insects, and can also be a pathogen for insects and mammals ^44^. Overall, environmental-associated bacteria have already been associated with dog skin microbiota and are to be expected, since dogs constantly interact with the environment ^27^.

Regarding *Stenotrophomonas* in human microbiota studies, Flores *et al*. found that this genus was enriched in atopic dermatitis patients that were responders to emollient treatment ^45^. However, previous studies on this skin disease found *Stenotrophomonas maltophila* associated to the disease rather than *Stenotrophomonas rhizophila* ^46^. *Achromobacter xylosoxidans* has been mainly associated with different kind of infections, as well as skin and soft tissue infections in humans ^47^. However, both dogs included in this pool were healthy and with representatives of both genus/species, a fact that reinforces the need to study the healthy skin microbiome at the species level before considering some species pathogenic. The other abundant bacteria detected on dog skin have been isolated in very different scenarios: *Sanguibacter keddieii* from cow milk and blood ^48, 49^; and *Glutamicibacter arilaitensis* (formerly *Arthrobacter arilaitensis*) is commonly isolated in cheese surfaces ^50, 51^.

In general, we obtained taxonomy assignment down to species level with both the full-length 16S rRNA gene and the 16S-ITS-23S of the *rrn* operon, although it was not always feasible due to: i) high similarity of the marker chosen within some genera, especially for the 16S rRNA gene; ii) an incomplete database; and iii) sequencing errors. In the light of these results, for an increased resolution at the species level, the 16S-ITS-23S of the *rrn* operon would be the best choice. At the expenses of an increased taxonomic resolution, we could have missed few bacterial species due to unlinked *rrn* genes. While in host-associated environments (e.g. gut) bacterial species with unlinked *rrn* genes are rare, if profiling natural environments (e.g. soil) this approach may be missing a significant proportion of the diversity ^52^. So far, this genetic marker does not have as many complete and curated databases as 16S rRNA gene. If choosing to use 16S-ITS-23S genetic marker, we could add some filtering steps to filter out the “wrongly assigned taxonomy” and have more reliable taxonomic results, both using the alignment block length and the AS score using Minimap2.

Other gene-marker strategies have been further described to profile the microbiota with Nanopore sequencing. For example, sequencing 16S rRNA genes by Intramolecular-ligated Nanopore Consensus (INC-seq) ^15, 53^ that allowed retrieving corrected full-length 16S rRNA genes. Another approach would be sequencing the cDNA from size selected small subunit rRNAs that allows retrieving many 16S rRNA genes using a primer-free approach ^54^. However, these alternative strategies have been applied to the 16S rRNA gene that has a limited taxonomic resolution within some genera. Recently an approach using unique molecular identifiers (UMIs) for obtaining corrected full-length *rrn* operon has been applied to characterize a mock community ^55^. The characterization of full-length ribosomal operons by the UMI approach has the potential to expand databases to make them more comprehensive with higher taxonomic resolution.

Further studies should be aiming to obtain reads with higher accuracy, either using consensus methods or applying new developments (new techniques, new basecallers or new R10 pores, etc.). Studies comparing marker-based strategies with metagenomics will determine the most accurate marker for microbiota studies in low-biomass samples.

## Data availability

### Underlying data

The datasets analyzed during the current study are available in the NCBI Sequence Read Archive, under the Bioproject accession number PRJNA495486.

### Extended data

All the supplementary data has been added in an OSF repository (doi: http://doi.org/10.17605/OSF.IO/8MYKV) ^22^. We provide here a complete list:

-  Supplementary Table 1. Primer sequences for amplifying the full-length 16S rRNA gene and 16S-ITS-23S of the *rrn* operon.

-  Supplementary Table 2. Samples included in the study, run summary and quality control results. *For mock communities, the mock DB was used. For complex communities, the *rrn* DB.

-  Supplementary Table 3. Taxonomic assignments table of each sample with the different approaches.

-  Supplementary Table 4. De novo results obtained with canu and their taxonomic assignment using BLAST.

-  Supplementary Figure 1. Photo of the agarose gel electrophoresis of some of the samples.

-  Supplementary Figure 2. Main workflow overview. Detailed bioinformatics workflow can be found in Supplementary File 1.

-  Supplementary File 1. Bioinformatics workflow used for the mapping approach.
