# Supplementary material for: Microbiota profiling with long amplicons using Nanopore sequencing: full-length 16S rRNA gene and the 16S-ITS-23S of the  rrn operon
Source: F1000Res. 2019 Aug 1;7:1755. Originally published 2018 Nov 6. [Version 2] doi: 10.12688/f1000research.16817.2 (PMC6372929; doi:10.12688/f1000research.16817.2)
Supplement: Peer review history

# Peer review: Reviewer response for version 2

**DOI:** 10.5256/f1000research.21950.r51954

**Published:** 2019-09-20

**Authors:** Lee J. Kerkhof

**Affiliations:** 1 Department of Marine and Coastal Sciences, Rutgers University, New Brunswick, NJ, USA

The authors have begun to put their findings into context with prior studies in the literature using MinION and rRNA genes or operons.   However, I disagree with their conclusions that the primers used for rRNA operon amplification need improvement rather than the choice of software and database.  Specifically, Cuscó *et al.* report that mock databases containing only those targets in the mock community can correctly assign OTUs using WIMP and MiniMap2 (Fig 3 and 4).  While larger databases such as a rRNA operon database or the NCBI Refseq database incorrectly assign taxa (from 12-55% of reads).   This may be true for the software they employed.  However, other studies have used QIIME, BLASTN, Centrifuge, and Discontinuous MegaBLAST to identify OTUs for the MinION platform for 16S rRNA genes or ribosomal operons.  I think an acknowledgement that these various software packages have also been employed to analyze the MinION reads in the scientific literature would benefit the F1000 readership.  

 Especially since the authors indicate that our study (Kerkhof *et al*., 2017 ^1^) supports their findings which is incorrect.  We actually found the databases which provided the most number of OTU calls for our raw reads were the NCBI 16S rRNA database (>18K entries) and the ARB/Silva NR99 database (>645K entries).  The intermediate size databases (Greengenes and RDP) did not work as well using Discontinuous Megablast.  I suspect the issue with incorrect OTU calling stems more from the software used to screen the database than from the primers or the size of the database being used.  

 A brief discussion of variability resulting from different PCR amplification reagents and what might be considered a best practice for data analysis for future studies would be helpful.

 Other Specific Comments:

1. Figure 3 has the calculated target gene concentration “Reference” on the right side of the heat map while Figure 4 has the “Reference” on the left side.  This makes it hard for the reader to figure out what should be for the WIMP or MiniMap2 results as a basis for comparison.

I confirm that I have read this submission and believe that I have an appropriate level of expertise to confirm that it is of an acceptable scientific standard, however I have significant reservations, as outlined above.

## References

[1] : Profiling bacterial communities by MinION sequencing of ribosomal operons. *Microbiome*.2017;5(1) : 10.1186/s40168-017-0336-9 10.1186/s40168-017-0336-9 28911333PMC5599880

# Peer review: Reviewer response for version 1

**DOI:** 10.5256/f1000research.18384.r43567

**Published:** 2019-02-21

**Authors:** Lee J. Kerkhof

**Affiliations:** 1 Department of Marine and Coastal Sciences, Rutgers University, New Brunswick, NJ, USA

F1000 Research Cuscó et al. ( https://doi.org/10.12688/f1000research.16817.1) 

**Comments to the authors:**

 The manuscript describes a study assaying 2 mock bacterial communities or 2 complex skin microbiome samples from dogs (chin or dorsal back) using both near-full length 16S rRNA genes and near-full length rRNA operons with the Oxford Nanopore MinION. The authors employ a library preparation method generating either 16S amplicons (1400 bp) or rRNA operons (4500 bp) including barcoding with a 1D ligation/sequencing kit and FLO-MIN 106 cells. The data analysis pipeline utilized Albacore basecalling, near-full length amplicon size selection, and screening by What’s in my Pot (WIMP) and Minimap2 against both NCBI and rrn databases. The authors demonstrate increased resolution at the species level with longer reads, that there can be large losses of raw sequence reads by size selection for rrn amplicons in their hands, and that the data analysis software and database can influence the results of MinION bacterial community analysis. 

 It would have been very helpful for the authors to put these findings into context with other papers in the literature using MinION and rRNA genes. For example, their results support what others directly sequencing near-full length16S amplicons (e.g. Shin *et al.* (2016 ^1^), Mitsuhashi *et al. *(2017 ^2^), and Benitez-Paez *et al.* (2016 ^3^)) or rRNA operons (e.g. Benitez-Paez *et al.* (2017 ^4^), Kerkhof *et al.* (2017 ^5^)) have shown in mock communities or complex samples with respect to species-level resolution. Additionally, the screening of MinION reads with different 16S rRNA databases has also been described in the supplementary figures of Kerkhof *et al.* (2017 ^5^). Likewise, an acknowledgement of the various software packages that has been employed to analyze the MinION reads in the scientific literature would benefit the readership. It appears that QIIME, BLASTN, Centrifuge, LAST aligner, Discontinuous MegaBLAST, WIMP, and MiniMap2 have all been used to identify OTUs for the MinION platform for 16S rRNA genes or rrn operons. As the authors have shown, the software/database being used can be very influential in the results of MinION screens and a synopsis of what they have found in context with other investigators (% bacterial assignment vs. % error) may point to a best practice for future studies.  

**Other Specific Comments:**

1. **Page 3:** I find it awkward/confusing to indicate the number of operons per microorganism per microliter here for the mock communities. Bacteria generally have 1-15 ribosomal operons in their genomes. I think it is clearer to just indicate the number of target rRNA operons is 10^3-10^6 for this particular DNA mixture.
2. **Page 3:** The barcoding expansion pack (EXP-PBC001) requires that the primers contain overhangs attached to the rRNA primers. This is not mentioned by the authors. Did they put overhangs on 27F/1492R/2241R? If so, the first round of target amplification may be affected by the presence of these overhangs. This should be indicated.
3. **Page 4:** The authors clearly show the danger of performing PCR and only characterizing the amplification product by Qubit fluorescence. If they had done agarose gels on the PCR reactions, they may have detected the short amplification products in their initial rrn operon reactions. Furthermore, these short reads are preferentially ligated using the SQK-LSK108 sequencing kit since there are more picomole ends. This best practice of visualizing PCR amplifications for size determinations before sequencing should be explicitly stated.
4. **Page 4:** I am a little confused by the 0.5 nM notation for PCR product in the barcoding reaction. If the authors used 50 microliter reactions, did they put 25 ng of 1st round PCR product in their barcoding reactions for a 15 cycle amplification? Can the authors just state the mass of DNA used to barcode? Secondly, Table 2 indicates BC1, BC2, and BC3 were not used. Was there a reason these barcodes were not utilized?
5. **Page 6:** Stating that the rrn operon profiling was more biased probably because of the lower sequencing depth does not recognize that others have not reported comparable bias or that it is probably a reflection of their potentially compromised amplification efficiencies. This conclusion should be viewed with caution, given the amplification issues noted above.
6. **Page 11:** The running of shorter (1500 bp) and longer (4500 bp) libraries on the same flow cell at the same time should enrich for the shorter reads. The MinION uses electrophoresis to move DNA molecules through the pores and smaller fragments should mobilize easier.

I confirm that I have read this submission and believe that I have an appropriate level of expertise to confirm that it is of an acceptable scientific standard, however I have significant reservations, as outlined above.

## References

[1] : Analysis of the mouse gut microbiome using full-length 16S rRNA amplicon sequencing. *Scientific Reports*.2016;6(1) : 10.1038/srep29681 10.1038/srep29681 27411898PMC4944186

[2] : A portable system for rapid bacterial composition analysis using a nanopore-based sequencer and laptop computer. *Scientific Reports*.2017;7(1) : 10.1038/s41598-017-05772-5 10.1038/s41598-017-05772-5 28720805PMC5516037

[3] : Species-level resolution of 16S rRNA gene amplicons sequenced through the MinION™ portable nanopore sequencer. *Gigascience*.2016;5: 10.1186/s13742-016-0111-z 4 10.1186/s13742-016-0111-z 26823973PMC4730766

[4] : Multi-locus and long amplicon sequencing approach to study microbial diversity at species level using the MinION™ portable nanopore sequencer. *GigaScience*.2017;6(7) : 10.1093/gigascience/gix043 10.1093/gigascience/gix043 PMC553431028605506

[5] : Profiling bacterial communities by MinION sequencing of ribosomal operons. *Microbiome*.2017;5(1) : 10.1186/s40168-017-0336-9 10.1186/s40168-017-0336-9 28911333PMC5599880

# Peer review: Reviewer response for version 1

**DOI:** 10.5256/f1000research.18384.r43564

**Published:** 2019-02-18

**Authors:** Kon Chu

**Affiliations:** 1 Department of Neurology, Seoul National University, Seoul, South Korea

The study compared the results of microbiota profiling using two different markers (16S rRNA and the rrn operon) and different classification methods. Because other reviewers have already made comprehensive reviews and comments including several critical points, I would like to add only a few minor points to the manuscript:

  

1. Figure 2: according to the text, *Actinomyces odontolyticus* was detected using the 16S rRNA gene, however, '0' in the figure can create confusion. It would be better to represent the number of copies of *Actinomyces odontolyticus* using more decimal places or adding a caption for this species.  
2. Figure 3a:
  - It would be better to change the figure (e.g. heatmap) to make it easier for readers to recognize under-represented and over-represented bacteria. *Listeria monocytogenes* also seems under-represented in the analyses using the mock database and rrn database, and the corresponding sentence in Page 7 may be changed.
  - Include the classification method (WIMP, minimap2) along with the name of the database, as in figure 4, to allow general readers to more easily match the methods and the database.
3. In the last paragraph of page 7, it seems that the criteria of the percentage of wrongly assigned species for the rrn operon are different from that for the 16S rRNA gene.
4. Table 3: I suggest making a caption for the difference between 'Staphylococcus' and 'Other Staphylococcus'.
5. If the authors would like to insist on better resolution by using the rrn operon, they need to demonstrate the data of the analysis using multiple species including species that tend to be under-represented or over-represented. 

I confirm that I have read this submission and believe that I have an appropriate level of expertise to confirm that it is of an acceptable scientific standard, however I have significant reservations, as outlined above.

# Peer review: Reviewer response for version 1

**DOI:** 10.5256/f1000research.18384.r43563

**Published:** 2019-02-08

**Authors:** Amanda Warr

**Affiliations:** 1 Roslin Institute, Royal (Dick) School of Veterinary Studies, University of Edinburgh, Edinburgh, UK

Cusco *et al. *evaluate methods for long read sequencing and classification of marker genes from microbial communities, both for mock communities of known microbial composition and complex communities from dog skin from two anatomical locations, chin and back. They find that long read sequencing of 16S and the *rrn *operon is sufficiently accurate to classify microbes and that *rrn *is more sensitive at the species level.

 This work demonstrates a valuable option for species identification from microbial samples using long reads, overcoming the current high error rate through covering a larger region. The work also highlights some of the issues that can arise from multiplexing amplicons of differing lengths when using Nanopore sequencing.

 Overall the paper is well written and detailed, however there are a few details I feel could be addressed:

1. **16S length reads in *rrn* barcodes: **Do you expect this to be entirely from barcode misassignment or were these shorter fragments produced during PCR? You state that the loss of *rrn* amplicons during the length trimming step was probably due to over-representation of 16S amplicon on the flow cell, and most of the reads lost were roughly 16S amplicon sized - are you suggesting that there are large numbers of 16S reads that are assigned to *rrn* barcodes after 2 rounds of demultiplexing? Are these shorter reads actually whole 16S amplicons or fragments of *rrn*?
2. **Expected sensitivity given read count** **:** The authors state that failure to detect the less abundant species from the mock community in the *rrn *dataset was "probably" due to their being fewer reads. As the proportions of the species in the mock samples are known, theoretically what total number of reads would be necessary to detect the less abundant species? Given the number of rrn reads obtained, did the authors detect as many species as they would expect to detect and what is the minimum total number of reads they would need to be likely to detect the lowest abundance species?
3. **Differences in classification methods** **:** Differences in classifications between the mock community database/rrn database and the NCBI database may be attributable to differences in the tools, with minimap2 being used for the mock and *rrn *databases and WIMP (based on centrifuge) being used for the NCBI database. My understanding is that the authors are mainly interested in classifications from different databases rather than differences between methods. While the authors do not directly compare the classification results between these different methods in text, some of the figures appear to imply that these results are directly comparable (e.g. Figure 3a). It would be useful if either all three databases were used with a single method (for example, using centrifuge with all three databases) or if these were at least more obviously separated or marked as coming from different classification methods in the figures.
4. **Classification rates against NCBI: **The authors should further discuss ways to improve the classification rates, will the biggest improvements come from reduced error rate, better classification tools, improving species representation in databases? The authors conclude that in the future we should aim to improve accuracy, but one of the main results here is that sequencing the full 16S/ *rrn *overcomes the problem of the current error rate - perhaps highlight benefits such as improved barcode assignment and emphasise that while this works well classification against a large database would likely improve with increased accuracy. The authors also conclude that *rrn *offers higher resolution at species level, however I suspect that currently more species have 16S sequences in databases than *rrn*.

Additionally, I have a few minor corrections mainly around small grammatical errors and figure/table modifications:

- Page 5: Paragraph beginning "To assign taxonomy...", change "to strategies" to "two strategies". Also I would change the last sentence on the page to say "some of the reads excluded were the expected length of the 16S rRNA gene rather than the *rrn* operon". Figure 1 should also be labelling Albacore as the basecaller.
- Page 6: change "would allow us determining" to "would allow us to determine".
- Page 11, column 2, line 2: change "associated to" to "associated with".
- Figure 3a would benefit from separating the reference bar from the other bars or adding this bar to the other two plots (currently it is grouped with Mock database, but it is also relevant to the *rrn* database and the NCBI database).
- Figure 4 text is quite difficult to read.
- Table 2: the title of the final column isn't clear. Is this the % of reads that pass the quality filters before chimera detection? Could another column be added showing number of reads that pass this filter? 
- Figure 5: there are several different colours of 0 in this heat map?

In the conclusion the authors have suggested ways to improve accuracy of this method in the future, I would add the R2C2 method (Volden *et al.*, 2018 ^1^) as an option to improve consensus accuracy here also, while designed for cDNA it could be applied to fragments of genomic DNA.

I confirm that I have read this submission and believe that I have an appropriate level of expertise to confirm that it is of an acceptable scientific standard.

## References

[1] : Improving nanopore read accuracy with the R2C2 method enables the sequencing of highly multiplexed full-length single-cell cDNA. *Proceedings of the National Academy of Sciences*.2018;115(39) : 10.1073/pnas.1806447115 9726-9731 10.1073/pnas.1806447115 PMC616682430201725

# Peer review: Reviewer response for version 1

**DOI:** 10.5256/f1000research.18384.r43566

**Published:** 2019-02-04

**Authors:** Rasmus H. Kirkegaard

**Affiliations:** 1 Center for Microbial Communities, Department of Chemistry and Bioscience, Aalborg University (AAU), Aalborg, Denmark

**Title:**

 "Microbiota profiling with long amplicons using Nanopore sequencing: full-length 16S rRNA gene and whole rrn operon".

**Summary of the key results:**

 The study demonstrates the use of nanopore sequencing for characterising low biomass samples with high levels of host DNA using a primer-based approach targeting the entire 16S rRNA gene or the 16S rRNA gene and the 23S rRNA gene.

 Furthermore, it evaluates the ability of these methods in the context of known references using mock communities and a pure culture using both the WIMP software and a custom mapping-based approach.

 The study demonstrates that nanopore sequencing can give accurate classifications even at the current level of error rate if the reference database contains the right sequences. The study also shows how sequencing the longer fragment spanning both the 16S rRNA and 23S rRNA genes improves the taxonomic classification when the database contains a matching sequence.

**Is the work clearly and accurately presented and does it cite the current literature?**

 The study mentions that the classification methods rely heavily on reference databases so it would be relevant to include citations for papers with methods for producing new reference sequences for both 16S rRNA and the longer fragment in the discussion (metagenomics, artificial long reads, primer free methods). Methods for improving read accuracy are also mentioned as important but the only methods mentioned are future upgrades from the company, relevant existing literature is not included (INC-seq, UMIs etc.). The study concludes that sequencing the entire “rrn operon” would be the best choice but it would be relevant to compare the size of current databases for the 16S rRNA gene versus the rrn operon. The presence of conserved sites for designing better primers is also extremely important but not discussed. Furthermore, there is evidence that quite a few organisms have unlinked rRNA genes, which will thus be missed by a full operon approach.

 Citations are also needed for bioinformatics tools for both processing and visualisation of the data.

**Is the study design appropriate and is the work technically sound?**

 The study uses mapping to a reference database to point out that the sequences can get genus- and species-level classification. However, the method will always report a genus and a species even in the absence of the correct sequence in the reference database as indicated from the sequencing of the *S. pseudintermedius* pure culture with the “rrn” method. It will be important to simulate the impact on the results when there is no closely related sequences in the database. This could be done by removing all reference sequences within the Gammaproteobacteria and mapping the HM-783D to the modified database and monitor where the reads end up. It would also be helpful if there was a way to distinguish between reads that have the “correct” match and reads that just happen to map because the 16S rRNA gene is extremely conserved. Something similar would be relevant for the EPI2ME workflow but as the authors cannot control the reference database, it is probably not feasible. One of the advantages of the mock communities should be information about the copy numbers for the rRNA genes but there is no information on this included in the study and how it affects the results.

**Are sufficient details of methods and analysis provided to allow replication by others?**

 The methods section lacks information about what happens after mapping the reads. How are the figures generated, what software is used, etc.? It would also be helpful if the specific scripts/commands used to run the bioinformatics analysis were available.

**Figures:**

**Figure 1: bioinformatic workflow:**

 The figure gives a decent overview of the bioinformatics processing but seems to miss the visualisation tools used. The main role of Albacore is basecalling the raw data not just demultiplexing. The figure could be improved further if you include the wet lab part of the work, so it becomes clear why the demultiplexing step is included and where the raw data comes from. A mapping step is integrated in the chimera detection (removal?) workflow but it might be better to omit mentioning mapping in that step as it can be confusing that the figure has two mapping steps in a row.

**Figure 2: heatmap mock community:**

 The caption needs to explain what the numbers represent e.g. percentage of sequenced reads/mapped reads. It would be great if the heatmap included the “true” composition of the mock community for comparison. Copy number for each organism in the mock would also be relevant to include in the figure. Since there are only two columns, it would be better to have the sample labels at the top and with horizontal text preferably with a name that makes it easier to interpret the figure.

**Figure 3a: stacked bar chart:**

 Even though stacked bar charts are very common it is not easy to read as they lack a common baseline for most of the values (See https://solomonmg.github.io/blog/2014/when-to-use-stacked-barcharts/ and https://peltiertech.com/stacked-bar-chart-alternatives/). I suggest that you use more of the heatmaps instead of introducing bar charts.

**Figure 3b: rarefaction curves:**

 It would be great if you could add a dashed line for the expected “true” value for the mock community.

**Figure 3c: WIMP tree:**

 This figure is quite complex to read. If the point with running both WIMP and a mapping-based approach with the two different amplicon types is to compare the methods, I suggest that you try to integrate the information better into one combined figure. This way you can help the reader to understand your message.

**Figure 4a: stacked bar chart+heatmap dog samples:**

 Remove the stacked bar chart.

**Figure 4b: stacked bar chart+heatmap dog samples:**

 Remove the stacked bar chart.

 Getting rid of the bar charts would allow for making a big heatmap with the data from Figure 4A and 4B combined. This way the reader can also compare the results from the two different sample sites. A naming system that makes it clearer that “_1” and “_2” are replicates would also help the reader interpret the figure. Presenting results aggregated at different levels, which could be included in one another is a bit confusing e.g., “ *Bacillus cereus*” could be included in “ *Bacillus*” which again could be included in “ *Bacillales*”.

**Figure 5: heatmap mock community contamination:**

 It is confusing that several cells in the heatmap have a value of “0” but with very different colours. Adding some meaningful labels with the contamination vs no contamination on the top could help the reader understand the figure without reading the caption.

**Tables:**

**Table 1: Primer sequences:**

 Fine but could be moved to supplementary.

**Table 2: Samples and QC:**

 Make headers easier to understand e.g. ”% seq 1st QC” could be “% of reads passing QC”, “Albacore pass” could just be “# reads after basecalling” etc. Where is the number after chimera detection?

 Add a column with data accession ID and move the table to supplementary then the sample names can also be expanded so the reader does not have to look to the bottom for an explanation of abbreviations. I suggest adding a column at the end with the number of reads mapping/classified for each sample so the reader know what fraction is included.

**Table 3: Pure culture comparison WIMP vs. mapping:**

 You need to make it clear in the table that *Staphylococcus pseudintermedius* is missing from the “rrn” database. As the paper mentions genus- and species-level classification as the target you may benefit from aggregating the values for *S. pseudintermedius* and *S. pseudintermedius* HKU10-03 as splitting this into strains makes it more confusing as your numbers in the text do not match the ones in the table.

**Supplementary Table 1:**

 It would be great to include the mock communities in this table as well.

I confirm that I have read this submission and believe that I have an appropriate level of expertise to confirm that it is of an acceptable scientific standard, however I have significant reservations, as outlined above.

# Peer review: Reviewer response for version 1

**DOI:** 10.5256/f1000research.18384.r40373

**Published:** 2018-11-19

**Authors:** Alfonso Benítez-Páez

**Affiliations:** 1 Microbial Ecology, Nutrition and Health Research Unit, Institute of Agrochemistry and Food Technology-Spanish National Research Council (IATA-CSIC), Valencia, Spain

Cusco and co-workers present an evaluation of both a mock community and the dog skin associated microbiota. The authors made use of the single-molecule Nanopore DNA sequencing technology and compared two different technical approaches by studying the nearly-full 16S rRNA bacterial gene and the nearly-full bacterial rRNA operon.

 In my opinion, this work represents an important advance regarding the application of nanopore technology in the field of microbiome research.

 The main strength of the work is its detailed technical description regarding the protocols for library preparation, sequencing and basecalling, that altogether facilitate the reproducibility. Moreover, the genetic data generated was properly deposited in a specialized database for public accession to whomever may want to replicate the analysis of long reads by similar approaches or new ones.

 The figure quality is good and the information disclosed by them is well accompanied with appropriate captions.

 Notwithstanding, I have some minor concerns about the work that should be clarified, at least for me:

1. The last paragraph of page 7 describes the level of reads correctly assigned to species level for the microbial isolate *Staphylococcus pseudintermedius*. However, some of the values cited in the text do not match, at least, explicitly in Table 3. So, the authors should revise this issue or better describe the information obtained.
2. The authors found that the study of a nearly-full 16S rRNA gene reflects in a better way the expected abundances of microbial species present in the mock community tested. This comparative analysis with regard to the results obtained by using the rrn operon should be accompanied by a linear regression analysis, declaring respective Pearson's "r" coefficients, that can measure more accurately the efficiency of both methods and better support the authors' observations and conclusions.
3. Additionally to the observed richness (observed species) and Shannon diversity, the authors could also include a microbial community evenness evaluation of reference and observed microbiome data from the different approaches evaluated in the study, so that additional conclusions could be addressed.
4. Given the issues with underrepresentation of "rrn" data as a consequence of mixing this type of synthetic DNA with nearly-full 16S rRNA amplicons, the authors should highlight this observation as a major issue of this approach and state a clear recommendation to avoid this type of multiplexing for future studies.
5. It is necessary to better describe the contamination issues described in the last paragraph of the results (page 9). I'm not sure if this cross-contamination came from re-utilization of a flowcell or if this came from contamination of the barcoded-primer, used during nested PCR, with amplicons/DNA from the mock community. In a similar manner, the estimation of 6% of contamination has to be explained in detail (species/proportions discarded or having been taken into account to calculate this percentage).

I confirm that I have read this submission and believe that I have an appropriate level of expertise to confirm that it is of an acceptable scientific standard.
